# Supplementary material for: High lumenal chloride in the lysosome is critical for lysosome function
Source: eLife. 2017 Jul 25;6:e28862. doi: 10.7554/eLife.28862 (PMC5526669; doi:10.7554/eLife.28862)

**Supplementary File 1 |** Sequences used for *Clensor* and I4^cLY^_A488/A647_ assemblies. Oligo **P**, Oligo **D1** and Oligo **D2** combine to form *Clensor*. Oligo I4^cLY^_A488_ and I4^cLY^_A647_ combine to form I4^cLY^_A488/A647_. The sequences in matching colors are complementary.


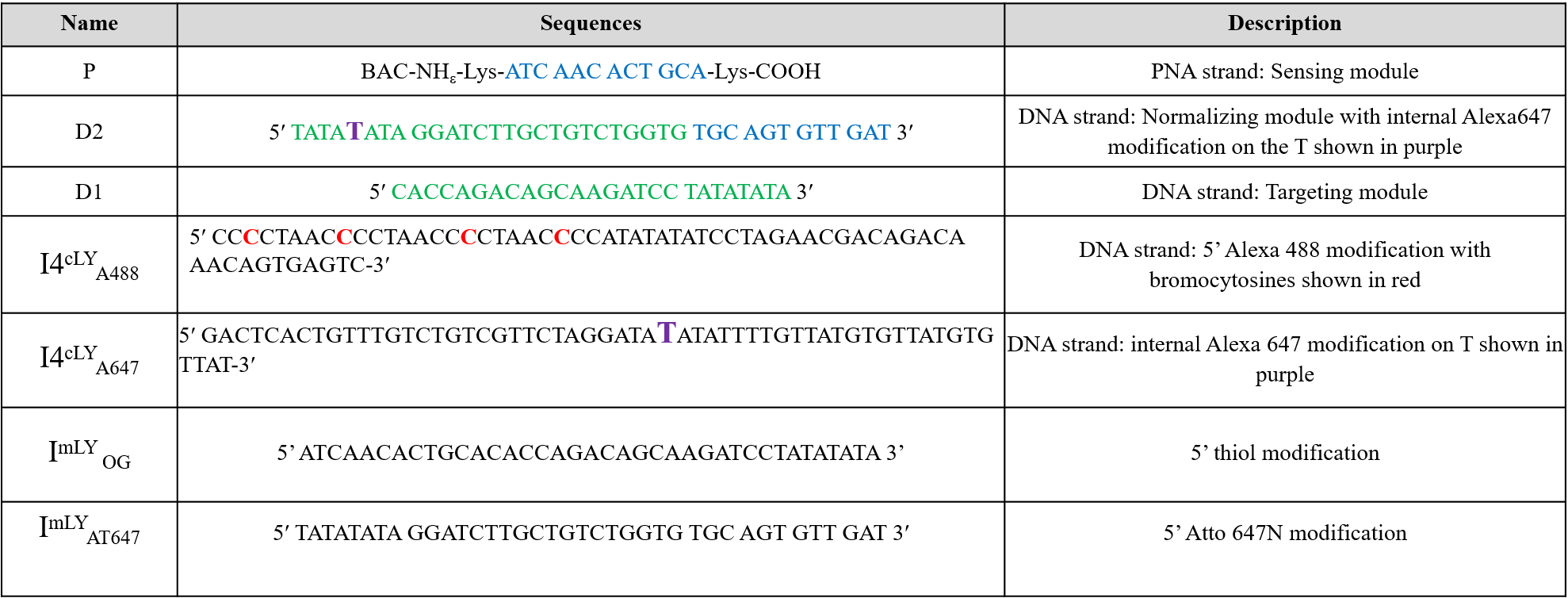

Supplement: Supplementary file 1. — Oligo P, Oligo D1 and Oligo D2 combine to form Clensor. Oligo I4cLYA488 and I4cLYA647 combine to form I4cLYA488/A647. The sequences in matching colors are complementary. DOI: http://dx.doi.org/10.7554/eLife.28862.023 [file elife-28862-supp1.docx]
