# Supplementary material for: High lumenal chloride in the lysosome is critical for lysosome function
Source: eLife. 2017 Jul 25;6:e28862. doi: 10.7554/eLife.28862 (PMC5526669; doi:10.7554/eLife.28862)
Supplement: Supplementary file 2. — DOI: http://dx.doi.org/10.7554/eLife.28862.024 [file elife-28862-supp2.docx]

**Supplementary File 2 |** Lysosomal storage disorders investigated in this study, their corresponding human genes and the *C.elegans* homologues.

| **Lysosomal storage disorder** | **Human Gene** | **C.elegans**  **homolog** |
| --- | --- | --- |
| Niemann Pick C | NPC1 | ncr-1 |
| Beta mannosidosis | MANBA | manba |
| Osteopetrosis | CLCN7 | clh-6 |
| Alpha mannosidosis | MAN2B1 | aman-1 |
| Mucopolysaccharidosis type VI  MPS (VI) | ARSB | sul-3 |
| Gaucher Disease | GBA | gba-3 |
| Niemann Pick A/B | SMPD1 | asm-1 |
| Neuronal ceroid lipofuscinoses  NCL | PPT1 | ppt-1 |
| Batten Disease | CLN3 | cln-3.2, cln-3.3, cln-3.1 |
